# Implementation and Clinical Impact of a Structured Clinical Pharmacy Service for Hospitalized Ileostomy Patients: A Retrospective Observational Study Using the RE-AIM Framework

**DOI:** 10.3390/pharmacy14030078

**Published:** 2026-05-27

**Authors:** Stefanie Hehenberger, Irene Lagoja

**Affiliations:** 1 Hospital Pharmacy, Klinik Ottakring, Montleartstraße 37, 1160 Vienna, Austria; 2 Vienna Doctoral School of Pharmaceutical, Nutritional and Sport Sciences, University of Vienna, Josef-Holaubek-Platz 2, 1090 Vienna, Austria

**Keywords:** implementation science, surgical stomas, ileostomy, pharmacy service, hospital, medication review, malabsorption syndromes, drug-related problems, high-output stoma, clinical pharmacy, RE-AIM framework

## Abstract

Patients with ileostomy face unique pharmacotherapeutic challenges, including altered drug absorption, yet structured pharmaceutical care for this population is rarely integrated into routine clinical practice. This retrospective monocentric cohort study evaluated the reach, effectiveness, adoption, implementation, and maintenance of a structured ileostomy-specific clinical pharmacy service using the implementation science-based RE-AIM framework at a 1245-bed teaching hospital in Vienna, Austria. Sixty-two patients (54.8% male; median age of 65.5 years) were included, receiving a median of 11 medications. A total of 324 drug-related problems (DRPs) were identified, of which 202 (62.3%) were classified as stoma-specific drug-related problems (SDRPs), representing a predefined subgroup of DRPs associated with ileostomy-related pharmacotherapy challenges. This distinction enabled separate evaluation of the clinical relevance of stoma-specific pharmaceutical interventions. The implementation rate was significantly higher for SDRP-related interventions than for general DRP recommendations (92.0% vs. 63.9%; *p* < 0.001), with no significant interdepartmental differences observed in either DRPs (*p* = 0.137) or SDRPs (*p* = 0.071). Patients with high-output stoma (HOS) had significantly more SDRPs than those without (*p* < 0.001), while no differences were observed in general DRPs. The service demonstrated wide adoption, high interprofessional acceptance, full protocol fidelity, and continuous implementation over 30 months. The findings provide implementation evidence that may support healthcare decision-makers, hospital administrators, and policy stakeholders in establishing and sustaining structured clinical pharmacy services for ileostomy patients in Austria and similar healthcare settings.

## 1. Introduction

In patients with an ostomy, anatomical alterations and postoperative physiological changes can significantly affect drug absorption. Key determinants include stoma location, residual bowel length, mucosal integrity, and the underlying disease. Particularly in the early postoperative period, increased gastric secretion and accelerated intestinal transit may reduce contact time with the intestinal mucosa, thereby limiting drug absorption [1,2]. Functional absorptive surface area appears to be the most critical determinant, which may explain the higher prevalence of absorption-related challenges in patients with an ileostomy compared with those with a colostomy [3,4,5].

These pathophysiological changes increase the risk of drug loss, reduced efficacy, and treatment failure, highlighting the need for stoma-specific medication optimization and clinical pharmaceutical assessment [5,6,7].

Despite this need, pharmacists are rarely involved in the routine care of ileostomy patients. Contributing factors include limited awareness of ileostomy-specific pharmacotherapeutic challenges, insufficient infrastructure for interprofessional collaboration, and the additional workload associated with specialized care. Furthermore, evidence on drug therapy optimization after stoma surgery remains scarce [8], and systematic knowledge regarding typical drug-related problems (DRPs), intervention strategies, and their practical implementation in clinical pharmaceutical care remains limited. The identification and classification of DRPs are core components of clinical pharmacy and are essential to patient counseling, quality assurance, professional development, and evaluation of pharmaceutical interventions [9,10].

Despite growing recognition of patient-centered clinical pharmacy practice in Europe, implementation of specialized clinical pharmacy services remains heterogeneous, particularly in Austria and other Central and Eastern European countries [11,12,13,14]. While medication reviews are widely established in hospital pharmacies, structured disease-specific clinical pharmacy services remain limited and are available only in selected institutions [13,15]. Broader and more systematic implementation of such services has therefore been recommended to improve medication safety and quality of care [11,12,16,17].

Implementation science offers structured approaches to evaluating how healthcare interventions can be integrated into routine practice. The RE-AIM framework is widely used to assess five key dimensions influencing real-world impact: Reach, Effectiveness, Adoption, Implementation, and Maintenance [18,19,20]. The framework is particularly suited to evaluating complex, multidisciplinary interventions and has been successfully applied across diverse healthcare settings [20,21,22].

Although structured care pathways for ileostomy patients are recognized as essential, no studies have systematically evaluated the implementation of structured clinical pharmacy services specifically designed for this patient group using an implementation science framework. Understanding not only the effectiveness but also the reach, adoption, implementation fidelity, and sustainability of such services in routine clinical practice is essential to developing scalable, evidence-based care models that improve medication safety and patient outcomes in this vulnerable population.

Therefore, this study aimed to evaluate the reach, effectiveness, adoption, implementation, and maintenance of a structured clinical pharmacy service for adult inpatients with ileostomy using the RE-AIM framework, assessing patient coverage, clinical impact (including drug-related and stoma-specific problems), organizational uptake, fidelity to protocol, and sustainability in routine hospital practice.

## 2. Materials and Methods

### 2.1. Study Design and Framework

This retrospective, monocentric cohort study evaluated the implementation and impact of a structured clinical pharmacy service for ileostomy patients, guided by the RE-AIM framework. RE-AIM was used both as a planning tool and as the primary reporting structure to capture clinical outcomes and implementation processes at patient, team, and organizational levels [18,19]. This study was conducted at a 1245-bed teaching hospital in Vienna, Austria, between December 2022 and June 2025. Reporting followed the STROBE recommendations for observational studies (see Supplementary File S1 for the completed checklist). Approval was obtained from the Ethics Committee of the City of Vienna (MA15-EK25-102-VK).

### 2.2. Setting and Intervention

Routine clinical pharmacy services at the institution primarily consist of level 3 medication reviews, defined as structured evaluations of a patient’s pharmacotherapy based on medication history, patient-reported information, and clinical data, including diagnoses, laboratory values, and medical records [23]. In addition, a standardized operating procedure (SOP) mandates structured clinical pharmacy involvement for all patients with an ileostomy due to the particular vulnerability of this population. This SOP-based service is applied irrespective of whether the ileostomy is newly created or pre-existing. It extends the standard level 3 medication review through a systematic assessment of stoma-specific pharmacotherapeutic challenges to identify stoma-specific drug-related problems (SDRPs). The structured SDRP assessment is arranged as decision trees to facilitate a systematic analysis and specifically covers six aspects: (1) dosage form, (2) t_max_, (3) medications influencing drug absorption, (4) stool volume, (5) oral fluid intake, and (6) relevant laboratory parameters [24].

The predefined clinical pharmaceutical workflow includes consultation within 48 h of hospital admission or after stoma creation, comprehensive medication analysis, identification and documentation of DRPs and SDRPs, recommendations to the medical team, follow-up, and evaluation.

Consultation requests are submitted via the institutional electronic hospital information system by physicians, stoma therapists, or nursing staff.

Based on this assessment, a written clinical pharmaceutical consultation note is generated and, whenever feasible, complemented by direct verbal communication with the responsible clinical team. Follow-up includes reassessment of stoma output, medication-related outcomes, laboratory parameters, implementation of recommendations, and identification of newly emerging problems or clinical improvements during inpatient stay.

**Figure 1:**
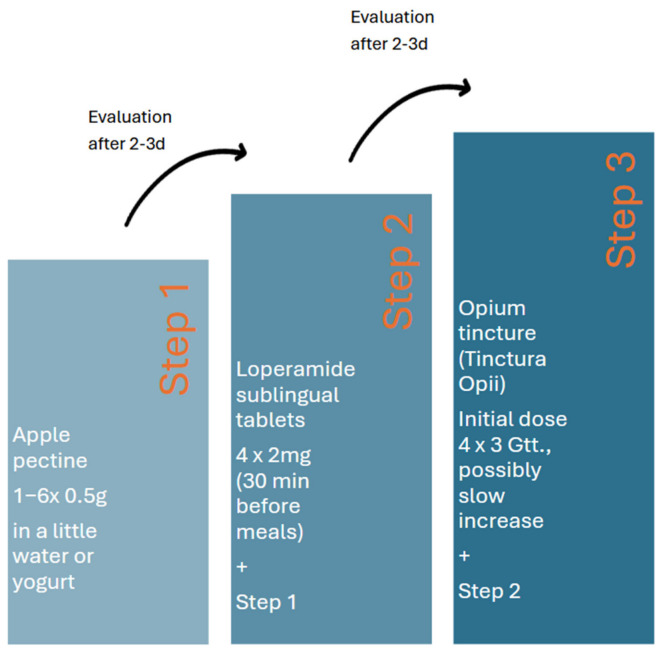
Treatment algorithm for stool volumes ≥ 1500 mL/d or very runny stools despite dietary measures without taking into account patient-specific factors such as kidney function and concomitant diseases [25].

For the pharmacological management of high-output syndrome (HOS), the SOP further includes a structured medication escalation algorithm (Figure 1), previously developed by a pharmacist-led expert panel [25]. This algorithm was developed because no official guidelines for the medical management of HOS exist and is applied according to the SOP to patients with a stoma output exceeding 1500 mL/day and/or very thin stool consistency.

### 2.3. Study Population and Eligibility

Patients were eligible if they were ≥18 years of age, had a newly created or pre-existing ileostomy, received routine clinical pharmacy services according to the institutional SOP, and had complete pharmacotherapeutic and clinical documentation. Patients were excluded if documentation was incomplete or if the pharmaceutical consultation process was not fully completed. All eligible patients treated during the study period were consecutively included to reflect real-world service delivery.

### 2.4. Data Collection

Data extraction was performed by a single trained clinical pharmacist to ensure standardized assessment. Structured electronic documentation templates and standardized data collection forms developed for routine clinical pharmaceutical care were used. Extracted variables were transferred into Microsoft Excel^®^ (Office Professional Plus 2016) for anonymization and analysis preparation. To enhance data consistency and completeness, all extracted data were cross-checked against the original medical records and pharmacy documentation prior to analysis. As the study was based on retrospectively collected routine clinical care data using established institutional documentation procedures, no separate pilot testing of the data collection forms was performed.

Information was obtained from electronic medical records, paper charts, pharmacy documentation, and laboratory systems. Extracted variables included ward of admission, clinical specialty, demographic data, all prescribed medications (active ingredient, product, and dosage form), primary diagnoses, comorbidities, clinical findings, indications for stoma creation, relevant laboratory parameters (e.g., electrolytes and renal function), and information from interprofessional discussions and patient interviews when available. Clinical outcomes were evaluated during inpatient stay until discharge or transfer. No standardized long-term post-discharge follow-up was performed as part of this retrospective evaluation.

### 2.5. DRP and SDRP Identification and Classification

For this evaluation, DRPs, SDRPs, and intervention types were conceptualized as qualitative indicators of process quality and system-level pharmaceutical care performance [9,10], as well as instruments for the systematic identification of key clinical themes and the most relevant problem areas in ileostomy pharmacotherapy. DRPs were defined as any event or circumstance involving drug therapy that actually or potentially interfered with desired health outcomes. SDRPs were defined as a predefined subgroup of DRPs directly related to ileostomy-specific pharmacotherapeutic challenges, including altered drug absorption, dosage form-related issues, high-output syndrome management, fluid and electrolyte disturbances, or accelerated gastrointestinal transit [24]. Thus, SDRPs represent a specialized subset of DRPs specific to the ileostomy population. Accordingly, individual patients could simultaneously present with both general DRPs unrelated to the ileostomy and SDRPs specifically associated with stoma-related pharmacotherapy challenges. DRPs and SDRPs were classified using a PCNE Classification V 9.114-based system adapted for ileostomy-specific pharmacotherapeutic issues (Supplementary File S2). Identified DRPs and SDRPs were documented together with the corresponding pharmaceutical interventions, which were categorized according to the predefined classification system.

### 2.6. RE-AIM Evaluation Framework

The evaluation was structured according to the five RE-AIM dimensions [18,19,26]:

- Reach: Proportion and representativeness of eligible ileostomy patients who received the clinical pharmacy service.
- Effectiveness: Identification and resolution of DRPs/SDRPs, acceptance rate of pharmaceutical interventions, and clinical outcomes (e.g., reduction in stoma output in HOS).
- Adoption: Proportion of hospital wards and clinical teams that used the clinical pharmacy service for ileostomy patients.
- Implementation: Fidelity to the SOP, consistency and intensity of service delivery and barriers/facilitators to implementation.
- Maintenance: Sustained service delivery over the 30-month study period and integration of the service into routine institutional practice.

### 2.7. Data Analysis

Descriptive statistics were performed using Microsoft Excel^®^ (Office Professional Plus 2016), and inferential analyses were conducted using IBM SPSS Statistics (Version 31). All data were anonymized prior to analysis. Normality was assessed using the Kolmogorov–Smirnov and Shapiro–Wilk tests, supplemented by visual inspection of histograms and Q–Q plots. As all variables deviated significantly from a normal distribution (*p* < 0.001), nonparametric tests were applied, as they do not require normality and are robust to skewed distributions.

To assess interdepartmental differences in the implementation of DRPs and SDRPs, patients were categorized according to the treating department. Departments with very small sample sizes (neurology, urology, trauma surgery, psychiatry, and pulmonology) were combined to ensure adequate group sizes for statistical analysis.

Between-group comparisons were performed using Kruskal–Wallis tests (e.g., ward comparisons), Mann–Whitney U tests (e.g., patients with vs. without HOS), and Wilcoxon signed-rank tests (paired implementation rates) [27,28]. Statistical significance was set to α = 0.05.

### 2.8. Quality Assurance and Bias Control

To ensure methodological rigor and enhance internal validity, several quality assurance and bias control measures were implemented throughout the study.

A central quality assurance element was the formal integration of the clinical pharmacy service into a binding institutional SOP [29]. This SOP predefined eligibility criteria, workflow processes, documentation standards, and the structured assessment of DRPs and SDRPs [30,31]. The use of a standardized level 3 medication review and a standardized medication analysis algorithm for patients with an ileostomy ensured uniform depth and scope of pharmacotherapeutic evaluation across all patients [23,24].

Furthermore, the therapeutic escalation algorithm for HOS provided predefined decision pathways, reducing variability in intervention strategies and limiting discretionary bias in treatment recommendations.

All medication analyses and DRP/SDRP classifications were performed by a trained clinical pharmacist with expertise in stoma care. While single-evaluator assessment may introduce observer bias, it simultaneously enhanced procedural consistency and minimized inter-rater variability. Documentation followed predefined categories to reduce subjective interpretation.

Data extraction from medical records adhered to structured templates, thereby limiting selective reporting and improving reproducibility [30,31].

## 3. Results

Results are presented according to the five RE-AIM dimensions to capture both clinical impact and implementation outcomes. A concise overview is provided in Table 1, while detailed findings are presented in the following subsections.

### 3.1. Reach

#### 3.1.1. Target Population and Service Coverage

A total of 62 medication analyses were evaluated over the 30-month study period, representing all eligible ileostomy patients who received clinical pharmacy services according to the institutional SOP. The study population comprised 34 male (54.8%) and 28 female (45.2%) patients with a median age of 65.5 years (range: 25–96).

Indications for ileostomy included oncological indications in 24 patients (38.7%), inflammatory bowel disease in 3 patients (4.8%) and other reasons (ileus, protective ileostomy, etc.) in 35 patients (56.5%). Patients received a median of 11 medications (range: 3–22), with 74.2% of patients being classified as experiencing hyperpolypharmacy (≥10 medications). The distribution across clinical departments demonstrated broad reach over 11 departments (Table 2).

#### 3.1.2. Medication Characteristics

Retrospective evaluation of all prescribed medications (*n* = 482) showed that 23.4% were administered parenterally and 69.7% orally. According to the classification framework proposed by van der Linde [7], oral formulations were categorized as rapidly releasing formulations (non-critical), immediate-release formulations with potential absorption limitations in ileostomy patients (potentially critical), and modified-release or enteric-coated formulations with a high risk of impaired drug release or absorption (critical). Applying this framework to all prescribed medications, 174/482 products (36.1%) were classified as potentially critical and 83/482 products (17.2%) as critical.

Among the 202 substances associated with pharmaceutical interventions, pantoprazole (ATC A02BC02) was most frequently involved (*n* = 36; 17.8%), followed by trazodone (N06AX05; *n* = 12; 5.9%).

Key characteristics of the prescribed medications are summarized in Table 3.

### 3.2. Effectiveness

#### 3.2.1. DRPs/SDRPs

In total, 324 DRPs were identified, corresponding to a median of 4 DRPs per patient (range: 1–14). A Kruskal–Wallis test showed no significant differences between departments in the number of DRPs (H(4) = 4.00, *p* = 0.406). Of all DRPs, 202 (62.3%) were classified as SDRPs, representing stoma-specific pharmacotherapeutic issues requiring specialized pharmaceutical intervention. The overall implementation rate of 122 general DRP-related recommendations was 63.9% (78/122). Of the 202 SDRP-related interventions, 186 (92.0%) were accepted and implemented by the clinical teams. A paired Wilcoxon signed-rank test showed that the implementation rate was significantly higher for SDRPs than for other DRPs (Z = −4.47, *p* < 0.001). Kruskal–Wallis tests revealed no significant interdepartmental differences in implementation rates for DRPs (H(4) = 6.99, *p* = 0.137) or SDRPs (*p* = 0.071).

Pharmaceutical interventions related to SDRPs covered a broad range of categories (Table 4), with monitoring, change of dosage form, and prescription of a drug being the most frequent.

#### 3.2.2. HOS

HOS-related issues were the primary focus of pharmaceutical intervention in 31 of 62 cases (50.0%). Thirty patients (48.4%) with stoma output >1500 mL/day and/or very thin stool consistency were treated according to the predefined HOS pharmacotherapy escalation algorithm (Figure 1). One patient was transferred to another hospital before further interventions could be implemented. In line with the institutional SOP for ileostomy management, isotonic oral fluids were recommended as baseline therapy, with pharmacological treatment initiated when output exceeded 1500 mL/day or stool consistency was very thin. Within this algorithm, isotonic drinks were prescribed in 11 cases (36.7%); eight patients (26.7%) additionally received apple pectin; seven patients (23.3%) subsequently received loperamide; and in two of these cases (6.7%), further escalation with opium tincture was required. In two additional cases (6.7%), the algorithm could not be fully applied due to patient-specific reasons. Among patients who completed the predefined escalation regimen during hospitalization, stoma output decreased to <1000 mL/day within the documented inpatient observation period. No implementation issues or complications were documented during HOS management. No severe adverse events related to pharmaceutical interventions were documented in the available medical records; however, adverse events were not systematically assessed as a predefined outcome.

A Mann–Whitney U test (N = 62) showed no significant differences in the number of DRPs between patients with and without HOS (U = 506.00, z = 0.37, *p* = 0.714). In contrast, the number of SDRPs was significantly higher in patients with HOS (U = 719.50, z = 3.42, *p* < 0.001).

### 3.3. Adoption

The clinical pharmacy service for ileostomy patients was introduced throughout the hospital. During the study period, patients in 11 different clinical departments received clinical pharmaceutical care, demonstrating broad organizational acceptance. The service was integrated into routine care pathways through the institutional SOP, which mandates structured involvement of the clinical pharmacy for all ileostomy patients.

The acceptance rate of pharmaceutical interventions for SDRPs (92.0%) reflects strong engagement by clinical teams from multiple disciplines. Recommendations were communicated in writing and/or in person, facilitating interprofessional collaboration.

The service was integrated into multidisciplinary care teams including physicians, nurses, stoma therapists, dietitians, and clinical pharmacists, reflecting comprehensive organizational adoption of the structured care model.

### 3.4. Implementation

All eligible patients received the standardized intervention according to the institutional SOP indicating high adherence to the predefined protocol. The intervention comprised: level 3 medication analysis for all patients (100%) and systematic evaluation of absorption-relevant factors, gastrointestinal transit, and ileostomy output according to a structured medication analysis algorithm (100%).

Data extraction by a single trained clinical pharmacist ensured standardized assessment across all cases. The predefined clinical pharmaceutical workflow was consistently applied throughout the study period.

Despite the overall successful implementation of the structured clinical pharmacy service, several factors influenced its uptake, fidelity, and sustainability (Table 5).

The most significant barrier was the high time demand associated with comprehensive medication analysis in this complex patient population, which required integration into routine clinical workflows without dedicated additional resources. Interprofessional coordination across multiple disciplines necessitated substantial effort, as communication and documentation had to be carefully managed among physicians, nurses, stoma therapists, and pharmacists. Infrastructure limitations, including reliance on both electronic and paper-based records, occasionally delayed interventions and complicated data retrieval. Knowledge gaps among clinical staff regarding stoma-specific pharmacotherapy—particularly HOS management and absorption-related dosage form considerations—necessitated ongoing education and awareness raising. Finally, resource constraints, including limited staffing capacity and lack of dedicated funding for specialized clinical pharmacy positions, were identified as potential challenges for service expansion. Several factors contributed to successful implementation and sustained delivery. The standardized institutional SOP clearly defined roles, responsibilities, and workflows, providing institutional legitimacy and supporting high fidelity across clinical settings. Strong interprofessional collaboration, including active engagement from physicians, nurses, stoma therapists, and dietitians, facilitated rapid acceptance of pharmaceutical recommendations and integration into multidisciplinary care pathways. The high clinical relevance of stoma-specific interventions—addressing tangible patient needs such as HOS management and dosage form optimization—reinforced the perceived value of the service and encouraged adoption by clinical teams. The use of dedicated monitoring and decision-support tools, including stool documentation sheets, standardized measurement protocols, and predefined pharmacotherapy escalation algorithms, enhanced reproducibility, reduced variability, and ensured consistent high-quality care delivery.

### 3.5. Maintenance

The clinical pharmacy service was maintained continuously over the 30-month study period (December 2022 to June 2025), demonstrating sustained implementation. The service was embedded in institutional practice through the formal SOP, supporting long-term maintenance.

The SOP mandating structured clinical pharmacy involvement for all ileostomy patients represents institutionalization of the service into routine care pathways, supporting ongoing maintenance beyond the study period.

The development and application of standardized protocols for common issues (e.g., HOS management algorithm and isotonic beverage protocols) facilitated consistent service delivery and supported maintenance of the intervention over time.

This study established key quality indicators that can support ongoing maintenance and continuous quality improvement:

- DRP/SDRP identification rates;
- Intervention acceptance rates;
- Clinical outcome achievement (e.g., HOS resolution);
- Service coverage across clinical departments.

## 4. Discussion

This study evaluated the implementation and clinical pharmaceutical impact of a structured clinical pharmacy service for hospitalized ileostomy patients using the RE-AIM framework. Ileostomy patients were considered a high-risk subgroup due to altered gastrointestinal transit, impaired oral drug absorption, fluid and electrolyte instability, and the frequent occurrence of HOS, thereby requiring structured pharmacotherapeutic assessment beyond routine clinical pharmacy services. A high burden of DRPs and SDRPs was identified, with particularly high acceptance and implementation rates for stoma-specific pharmaceutical recommendations across multiple clinical departments. A key strength of this study is the application of the RE-AIM framework, which enabled a structured evaluation not only of clinical and pharmaceutical outcomes but also of implementation-related domains including Adoption, Fidelity, Reach, and Maintenance. This approach responds to increasing calls for stronger implementation science and theoretical frameworks in clinical pharmacy practice research [32,33].

### 4.1. Interpretation of Main Findings

In terms of Reach, the service covered all eligible adult ileostomy patients during the 30-month study period and included a broad spectrum of indications, age groups, and clinical specialties. This suggests that the intervention was not restricted to a narrow subgroup but integrated into routine care for a representative ileostomy population. The high prevalence of polypharmacy and hyperpolypharmacy highlights the pharmacotherapeutic complexity and vulnerability of this group, underscoring the need for specialized medication management.

With regard to Effectiveness, the high number of identified DRPs and the even higher proportion of SDRPs emphasize the clinical relevance of targeted clinical pharmaceutical analysis in ileostomy patients. A key finding of this study is that 62.3% of all identified DRPs were classified as stoma-specific, highlighting the unique pharmacotherapeutic challenges in ileostomy patients.

These findings are supported by the retrospective analysis of all prescribed medications in the present cohort, which revealed that 69.7% were administered orally and therefore potentially affected by altered gastrointestinal transit. Among all solid oral formulations, 36.1% were classified as potentially critical and 17.2% as critical with regard to their pharmacokinetic release characteristics according to the classification framework based on the European Pharmacopoeia (Ph. Eur.) and the categorization proposed by van der Linde [7]. The proportion of critical formulations aligns with the figures reported by Remane et al., further confirming a substantial and reproducible pharmacotherapeutic challenge [34]. This distribution underlines the significant proportion of medications in ileostomy patients that may be associated with impaired drug release or absorption and highlights the importance of systematic medication review and tailored pharmaceutical interventions in this vulnerable patient population. The higher proportion of stoma-specific DRPs (62.3%) observed in our study compared with mixed ostomy populations in the literature (45.1%) [8] can be attributed to our exclusive focus on ileostomy patients, who exhibit significantly higher rates of ostomy-related pharmaceutical interventions than colostomy patients due to reduced intestinal absorptive surface, accelerated transit time, and greater fluid and electrolyte losses [3,35].

### 4.2. Implementation Rate Differences

The implementation rate reported in the literature appears to have a wide range, varying between 52% and 100% [36]. The acceptance rate for DRPs observed in this study correlates with figures reported in similar settings in a large Austrian tertiary care hospital [37]. Notably, the overall acceptance rate of 92.0% for SDRP interventions exceeds those reported in many comparable clinical pharmacy studies.

The significantly higher implementation rate for SDRP-related recommendations compared with general DRPs (*p* < 0.001) suggests that stoma-specific interventions are perceived by prescribing physicians as particularly clinically relevant and actionable. This interpretation is consistent with previous findings indicating that recommendations of higher clinical priority and those tailored to specific patient needs are more readily implemented [38,39]. The specialized nature of SDRPs, their clear pathophysiological rationale, and their direct, tangible benefit for patients likely contributed to enhanced physician acceptance. Furthermore, acceptance of clinical pharmacist interventions is generally higher for specialized disease conditions and when pharmacists possess specific expertise in those areas [40]. Additionally, polypharmacy is generally associated with higher acceptance rates, possibly due to increased awareness of polypharmacy as a risk factor for drug-related problems [36]. Moreover, verbal communication of recommendations whenever possible likely contributed positively to acceptance [38,39,40]. Importantly, no significant differences in implementation rates were observed between departments, indicating consistent service quality across clinical settings. This contrasts with reports of specialty-dependent variability in acceptance rates and may reflect the standardizing effect of the institutional SOP and the continuous involvement of a dedicated clinical pharmacist across wards [40,41].

Taken together, the exceptionally high acceptance rate observed in the present study likely reflects the combination of highly specialized, indication-specific interventions, formal SOP integration, and strong interprofessional collaboration within established multidisciplinary care structures.

### 4.3. Role of HOS

HOS refers to accelerated stool passage with the secretion of large volumes of liquid stool through the stoma [42]. While an ileostomy typically produces 600–800 mL per day, a daily output of ≥1500 mL is generally considered characteristic of HOS, although no uniform definition exists [43,44]. HOS affects 14–24% of ileostomy patients [45] and is associated with significant risks, including dehydration, electrolyte disturbances, and acute kidney injury, making it the most common reason for readmission following stoma creation [42].

In the present study, HOS constituted the primary focus of pharmaceutical intervention in 50.0% of cases, substantially exceeding the reported prevalence of 14–24% in the general ileostomy population. This discrepancy may be explained by the high readmission rate associated with HOS-related complications, as patients with dehydration and electrolyte disturbances are likely overrepresented among hospitalized ileostomy patients. Consequently, the elevated intervention rate likely reflects the clinical complexity inherent in this patient subgroup rather than the true prevalence of HOS in the overall ileostomy population. A key finding was that while the total number of DRPs did not differ significantly between patients with and without HOS, patients with HOS exhibited a significantly higher rate of SDRPs. This novel observation highlights that HOS is predominantly associated with stoma-specific pharmacotherapeutic challenges rather than with the general DRP burden. Thus, HOS represents a distinct and clinically relevant domain requiring specialized pharmaceutical care.

Based on our findings, several key domains for structured HOS management emerged.

First, early recognition is critical. HOS may present with increased stoma output, altered consistency, weight loss, reduced urine output, or laboratory abnormalities. Structured documentation of stoma volume and urine output—both in hospital and after discharge—is essential to timely detection and intervention [6,7].

Second, differential diagnoses must be systematically excluded, including infectious enteritis, short bowel syndrome, intra-abdominal sepsis, disease recurrence, or medication-related causes [44].

Third, fluid management requires a pathophysiologically informed approach. Simply increasing oral intake may exacerbate losses due to limited absorptive capacity and sodium depletion [46,47,48]. Instead, oral intake should be restricted, hypotonic and hypertonic beverages avoided, and isotonic rehydration solutions preferentially used [46,49,50]. Evidence suggests that prophylactic isotonic supplementation may significantly reduce dehydration-related readmissions and improve renal outcomes [51].

Fourth, pharmacotherapy aims to reduce intestinal transit and fluid loss [52]. However, treatment remains largely off-label, and evidence-based guidelines are lacking [44]. In this context, structured, literature-based treatment algorithms—such as the SOP implemented in our institution—provide clinical guidance and standardization of care. The 100% success rate in reducing stoma output to <1000 mL/day using the predefined escalation algorithm suggests clinical effectiveness of structured pharmacotherapy protocols for HOS management. Although all patients completing the escalation pathway achieved the predefined output target during hospitalization, this finding should be interpreted cautiously given the small sample size, retrospective design, lack of comparator group, and absence of long-term follow-up data.

Finally, HOS management requires structured multidisciplinary collaboration. Given the complex metabolic and pharmacotherapeutic challenges involved, coordinated care among physicians, nurses, dietitians, stoma therapists, and clinical pharmacists is essential to preventing deterioration, optimizing fluid and electrolyte balance, and reducing readmissions [6,7,47,52,53,54].

### 4.4. Pharmaceutical Intervention Patterns

The most common intervention categories reflect typical gaps in care that arise from the specific characteristics of clinical pharmaceutical stoma care, which can be effectively addressed through structured pharmaceutical involvement:

- Monitoring both clinical parameters and laboratory values is essential to the early detection of fluid and electrolyte imbalances, changes in kidney function, and potential alterations or reductions in medication effectiveness [47]. It is critical to assessing the therapeutic impact of medications in patients with an ileostomy. Frequent monitoring helps guide necessary adjustments to optimize drug therapy and prevent complications related to absorption and efficacy.
- Changing the dosage form is particularly relevant for ileostomy patients; it is important to choose dosage forms that ensure rapid release of the active ingredient [55,56]. The prevalence of sustained-release (7.1%) and enteric-coated (10.2%) formulations among oral medications underscores absorption challenges in shortened bowel transit, where these may pass unabsorbed via stoma, compromising efficacy and necessitating dosage form changes [7].
- Therapeutic reorganization often concerned antimotility or antisecretory agents, which are key elements of HOS management and essential to stabilizing stoma output [52].

### 4.5. Adoption, Implementation, and Maintenance (RE-AIM Perspective)

Our findings also provide important insights into Adoption at the organizational and team level. The fact that patients from 11 different departments received the service, combined with the high acceptance rate of pharmaceutical recommendations, reflects broad interdisciplinary buy-in. The formal anchoring of the service in a hospital SOP appears to have been a key facilitator, as it clearly defines roles, processes, and expectations. This aligns with previous work showing that formalization and clear protocols promote the uptake of complex clinical services [41].

Regarding Implementation, this study shows that high fidelity to the predefined clinical pharmaceutical workflow is feasible in routine practice. All eligible patients received a level 3 medication analysis and structured SDRP assessment, and data were collected consistently by a trained clinical pharmacist. At the same time, the implementation process required considerable coordination efforts, including the systematic monitoring of stool volume, the clarification of differential diagnoses of high output, and close interprofessional communication. These observations illustrate that successful implementation of disease-specific clinical pharmacy services depends not only on clinical expertise but also on adequate resources, infrastructure, and collaborative culture.

From a Maintenance perspective, the continuous operation of the service over 30 months and its institutionalization through a binding SOP suggest that the intervention has moved beyond a pilot phase into sustained routine practice. The establishment of explicit quality indicators—such as DRP/SDRP detection rates, intervention acceptance rates, and HOS outcome achievement—provides a basis for ongoing monitoring and continuous quality improvement. This is particularly relevant in light of the broader European context, where the development and long-term stabilization of clinical pharmacy services remain challenging in many settings [57].

### 4.6. Strengths and Implications for Practice

A key strength of this study is the application of the RE-AIM framework, which enabled a structured evaluation of not only clinical outcomes but also adoption, fidelity, and sustainability. The consecutive inclusion of all eligible patients, the use of a structured medication analysis approach for individuals with an ileostomy, and the application of a standardized classification system for DRPs and SDRPs enhance the internal consistency and practical relevance of the findings. Furthermore, the integration of quantitative implementation outcomes (e.g., acceptance rates and departmental coverage) provides valuable information for decision-makers considering the introduction or expansion of similar services. Importantly, this study is based on real-world data derived from routine clinical practice, increasing its external validity and supporting the transferability of the findings to comparable healthcare settings.

For clinical practice, the results suggest that structured, stoma-specific clinical pharmacy services should be considered a core component of multidisciplinary ileostomy care. The high acceptance rate of recommendations indicates that clinical teams perceive clear added value in specialized pharmaceutical input. The HOS algorithm appears to be a practical and effective tool for standardizing and optimizing pharmacotherapeutic management in a high-risk subgroup. Hospitals with similar patient populations may benefit from adapting and implementing comparable structured care pathways, particularly when embedded within routine clinical workflows. The findings may support healthcare planners, hospital administrators, and funding stakeholders in evaluating the feasibility and value of implementing structured disease-specific clinical pharmacy services within multidisciplinary care pathways.

### 4.7. Limitations

This study has several limitations. First, its monocentric, retrospective design and relatively small sample size limits generalizability and precludes causal inference. The observed associations between the service and clinical outcomes may be influenced by unmeasured confounders, such as parallel improvements in surgical or nursing care.

Second, the absence of a control group without structured clinical pharmacy intervention means that we cannot directly quantify the incremental benefit of the service compared with usual care. In addition, the absence of standardized long-term follow-up limits conclusions regarding recurrence of HOS, long-term treatment adherence, and sustained clinical outcomes after discharge. Recurrence rates of HOS after discharge were not systematically documented and could therefore not be evaluated.

Third, data collection by a single pharmacist ensured consistency but may introduce observer bias, particularly in the identification and classification of DRPs and SDRPs. The service model was strongly dependent on a single trained clinical pharmacist, which may limit scalability and reproducibility in settings with fewer specialized personnel.

Furthermore, no severe adverse events related to pharmaceutical interventions were documented in the available medical records; however, adverse events were not systematically assessed as a predefined outcome.

Finally, patient-centered outcomes such as quality of life, readmissions, or long-term renal function were not systematically assessed and should be addressed in future studies.

### 4.8. Future Directions

Future research should aim to validate these findings in multicenter settings and different healthcare systems, ideally using prospective designs and comparison groups. It would also be valuable to examine cost-effectiveness, including potential reductions in length of stay, readmissions, or complications related to dehydration and electrolyte disturbances. The integration of patient-reported outcomes and qualitative evaluations of patient and staff experiences could further enrich the understanding of how and why such services work in practice.

## 5. Conclusions

This study suggests that a structured, ileostomy-specific clinical pharmacy service can be implemented with high fidelity, adopted across multiple hospital departments, and sustained over time. The use of an implementation science framework such as RE-AIM provides a robust basis for systematically planning, evaluating, and scaling such services within complex healthcare systems. Overall, these findings offer a practical framework for stakeholder engagement, resource planning, and the expansion of structured clinical pharmacy services within multidisciplinary surgical and medical care pathways.

## Figures and Tables

**Table 1:** RE-AIM evaluation of the structured clinical pharmacy service for ileostomy patients.

RE-AIM Dimension	Definition	Operationalization in This Study	Key Findings
Reach	Proportion and representativeness of the target population receiving the intervention	Inclusion of all eligible adult inpatients with new or pre-existing ileostomy over 30 months	62/62 eligible patients received the service (100% coverage); median age: 65.5 years (25–96); 74.2% hyperpolypharmacy (≥10 medications); broad distribution across 11 departments
Effectiveness	Impact of the intervention on relevant clinical and process outcomes	Identification and resolution of DRPs and SDRPs; intervention acceptance rates; HOS management outcomes	324 DRPs identified (median: 4/patient); 62.3% classified as SDRPs; overall DRP implementation rate: 63.9%; SDRP implementation rate: 92.0% (*p* < 0.001 vs. DRPs); 100% HOS output reduction to <1000 mL/day when full escalation applied
Adoption	Uptake of the intervention at organizational and team levels	Number and diversity of departments using the service; engagement of multidisciplinary teams; SOP integration	Service delivered across 11 departments; high interprofessional acceptance; formal institutional SOP mandating pharmacist involvement
Implementation	Fidelity, consistency, and intensity of service delivery; barriers and facilitators	Adherence to predefined workflow (level 3 medication review + structured SDRP analysis); documentation quality; intervention types	100% of patients received level 3 medication analysis and structured SDRP assessment; standardized workflow consistently applied; most frequent SDRP interventions: monitoring (35.6%), dosage form change (24.8%), and drug initiation (18.3%)
Maintenance	Sustainability and integration into routine practice over time	Duration of continuous service delivery; institutional anchoring; establishment of quality indicators	Continuous operation over 30 months; formalized through binding SOP; defined quality indicators (SDRP detection rate, intervention acceptance, and HOS outcome achievement); integrated into routine care pathways

**Table 2:** Characteristics of the included patients.

Characteristics of the Included Patients *n* = 62	Value
Gender	
Male [*n* (%)]	34 (54.8)
Female [*n* (%)]	28 (45.2)
Age, years [median (range)]	65.5 (25–96)
Number of medications [median (range)]	11 (3–22)
Polypharmacy ≥5 medications [*n* (%)]	15 (24.2)
Hyperpolypharmacy ≥10 medications [*n* (%)]	46 (74.2)
Indication for ileostomy	*n* (%)
Inflammatory bowel disease	3 (4.8)
Oncological indication	24 (38.7)
Other (ileus, protective ileostomy, etc.)	35 (56.5)
Ward distribution	*n* (%)
Department of General, Visceral, and Tumor Surgery	28 (45.2)
Center for Oncology and Hematology with Palliative Care	9 (14.5)
Medical Department with Nephrology and Dialysis	5 (8.1)
Medical Department with Gastroenterology, Hepatology, Endoscopy	7 (11.3)
Medical Department with Endocrinology, Rheumatology, and Acute Geriatrics	3 (4.8)
Department of Anesthesia, Intensive Care, and Pain Medicine	4 (6.5)
Neurology Department	1 (1.6)
Urology Department	1 (1.6)
Trauma Surgery Department	1 (1.6)
General Psychiatry Department	1 (1.6)
Medical Department with Pneumology	2 (3.2)

**Table 3:** Characteristics of the prescribed medications.

Prescribed Medications by Dosage Form, *n* = 482	*n* (%)
i.v. and s.c.	113 (23.4)
TTS	12 (2.5)
Oral	
Tablets	59 (12.2)
Film-coated tablets	104 (21.5)
Sustained-release tablets/capsules	34 (7.1)
Dragées/sugar-coated tablets	2 (0.4)
Granules/effervescent tablets	24 (5.0)
Enteric-coated forms	49 (10.2)
Hard capsules	9 (1.9)
Solutions, suspensions, drops	44 (9.1)
Chewable, sublingual, or orally disintegrating tablets	11 (2.3)
Other forms (inhalers, eye/ear drops, dermatological preparations, etc.)	21 (4.4)
**Substances with Most Frequent Pharmaceutical Interventions (ATC), *n* = 202**	***n* (%)**
Pantoprazole (A02BC02)	36 (17.8)
Trazodone (N06AX05)	12 (5.9)
Metamizole (N02BB02)	8 (4.0)
Loperamide (A07DA03)	8 (4.0)
Acetylsalicylic acid (B01AC06)	8 (4.0)
Hydromorphone (N02AA03)	6 (3.0)
Amlodipine (C08CA01)	5 (2.5)
Rosuvastatin (C10AA07)	5 (2.5)
Sertraline (N06AB06)	4 (2.0)
Potassium (A12BA01)	3 (1.5)

**Table 4:** SDRPs coded according to intervention type.

Description of Intervention, *n* = 202	*n* (%)
Prescription of a drug	37 (18.3)
Monitoring (e.g., laboratory or clinical follow-up)	72 (35.6)
Change of dosage form	50 (24.8)
Discontinuation of a drug	18 (8.9)
Change of route of administration	0 (0.0)
Change of active ingredient	4 (2.0)
Modification of dosage form	14 (6.9)
Dose adjustment or change in dosing interval	2 (1.0)
Optimization of administration (e.g., time of application)	2 (1.0)
Organizational and administrative support (e.g., adaptation to institutional drug list and logistics)	1 (0.5)
Information (for doctors, nursing staff, or patients)	2 (1.0)

**Table 5:** Summary of barriers and facilitators identified during implementation of the structured clinical pharmacy service, categorized by key implementation domains.

Category	Barriers	Facilitators
Time and Resources	High time demand for comprehensive medication analysis; limited staffing capacity; lack of dedicated funding	Standardized workflow reducing redundant efforts; integration into existing care processes
Interprofessional Collaboration	Coordination effort across multiple disciplines; variable engagement across wards	Strong multidisciplinary team structures; high acceptance of recommendations (92% for SDRPs)
Infrastructure	Fragmented documentation (electronic and paper-based); delayed data retrieval	Multiple data sources enabling comprehensive assessment; institutional SOP providing clear framework
Knowledge and Training	Knowledge gaps regarding stoma-specific pharmacotherapy; need for ongoing staff and patient education	High clinical relevance of interventions reinforcing learning; predefined algorithms reducing uncertainty
Sustainability	Single-pharmacist dependence; resource constraints limiting expansion	Institutionalization through binding SOP; established quality indicators for monitoring

## Data Availability

No new data were generated in this study. The analysis is based on retrospectively collected routine data from clinical pharmaceutical care.

## Supplementary Materials

The following supporting information can be downloaded at: https://www.mdpi.com/article/10.3390/pharmacy14030078/s1, File S1: STROBE checklist; File S2: Classification system.

## Abbreviations

The following abbreviations are used in this manuscript:

GI	Gastrointestinal
HOS	High-output syndrome
DRP	Drug-related problem
SDRP	Stoma-specific drug-related problem
SOP	Standard operating procedure
t_max_	Time to maximum plasma concentration
RE-AIM	Reach, Effectiveness, Adoption, Implementation, Maintenance
PCNE	Pharmaceutical Care Network Europe
ATC	Anatomical Therapeutic Chemical classification system
Ph. Eur.	European Pharmacopoeia
i.v.	Intravenous
s.c.	Subcutaneous
TTS	Transdermal therapeutic system
