# Supplementary material for: Implementation and Clinical Impact of a Structured Clinical Pharmacy Service for Hospitalized Ileostomy Patients: A Retrospective Observational Study Using the RE-AIM Framework
Source: Pharmacy (Basel). 2026 May 27;14(3):78. doi: 10.3390/pharmacy14030078 (PMC13306839; doi:10.3390/pharmacy14030078)
Supplement: Supplementary file 1 [file pharmacy-14-00078-s001.zip › Supplementary File S2- Classification system.pdf]

Supplementary File S2: Classification system

Classification of drug-related questions

M: Medication

AM 1: Prescription of an unavailable medication (not listed, not available, discontinued, etc.)

AM 2: Duplicate prescription (same active ingredient, same class of active ingredient)

AM 3: Transmission error

ANW: Application

ANW 1: Inappropriate application period (too long/too short)

ANW 2: Inappropriate time of use

DOS: Dosage

DOS 1: Overdose

DOS 3: Inappropriate dosing interval (daily dose OK, adjust frequency of administration)

IND: Indication

IND 1: No drug prescribed for existing indication

IND 2: Drug prescribed without apparent indication

IND 3: Therapy not in accordance with guidelines

KI: Contraindication

UAW: Adverse drug reaction (including potentially inappropriate medication - PIM)

WW: Interaction

WW 1: Pharmacokinetic interaction

WW 2: Pharmacodynamic interaction

IST: DRP in connection with stoma

Clinical pharmaceutical Interventions

1: Prescription (initiation) of a new drug

2: Discontinuation of a drug (pause and discontinuation)

3: Change of medication

4: Change of administration route (e.g., i.v. to oral)

5: Medication patient monitoring (checking certain lab values, medication levels)

6: Optimization of administration (time of application)

7: Dose adjustment (organ function, age, weight)

8: Information (physician, nursing staff, patient)

9: Organizational and administrative assistance (adaptation to institutional drug list, logistics inquiries)

10: Optimization of documentation (addition of dose, specification of mode of administration)

11: Other

IST 1: Prescription of a drug

IST 2: Monitoring (e.g. laboratory or clinical follow-up)

IST 3: Change of dosage form

IST 4: Discontinuation of a drug

IST 5: Change of route of administration

IST 6: Change of active ingredient

IST 7: Modification of dosage form

IST 8: Dose adjustment or change in dosing interval

IST 9: Optimization of administration (e.g., time of application)

IST 10: Organizational and administrative support (e.g., adaptation to institutional drug list, logistics)

IST 11: Information (for doctors, nursing staff, or patients)

Acceptance

1: Intervention leads to change

2: Intervention was considered by the physician

3: Intervention was rejected
